# The use of the Illumina FFPE Restoration Protocol to obtain suitable quality DNA for SNP-based CGH– a pilot study

**DOI:** 10.1186/1897-4287-10-S2-A85

**Published:** 2012-04-12

**Authors:** AN Hosein, S Cocciardi, J Jayanthan, S Song, P Simpson, G Chenevix-Trench

**Affiliations:** 1 The Queensland Institute of Medical Research, Brisbane, Australia; 2 The University of Queensland Centre for Clinical Research, Brisbane, Australia

## 

The use of formalin fixed paraffin embedded (FFPE) tissues for DNA analysis has been met with great difficulties due to the degradative effect this fixation, processing and storage has on DNA quality. The lack of a suitable protocol to enhance the quality of such degraded DNA has been a great hindrance to our ability to make full use of clinically annotated FFPE cancer tissue banks. In this report we have begun to investigate the effectiveness and limitations to Illumina’s recent platform for the restoration of DNA derived from archival FFPE tissues.

With the exception of select fresh frozen and blood samples, all DNA samples were extracted from FFPE tissues and restored according to Illumina’s protocol. The quality of the FFPE extracted DNA was then assessed by Illumina’s PCR-based quality control assay (QC-PCR) and the resultant DNA was subsequently run on Illumina’s SNP-based CGH chips. Chip call rates were largely used in order to determine the quality of a particular array. Overall, the chip data were highly reproducible as determined by comparing several technical replicate samples. FFPE-extracted and restored DNA performed well in comparison to DNA extracted from fresh frozen tumor and blood from the same patients, meeting the minimum standard for continuation of this platform. It should also be noted that the quality of the chip data was not appreciably enhanced by the use of sodium thiocyanate during the extraction of DNA from FFPE tissues. Moreover, chip quality was significantly lower, with regards to call rates and ‘quality’ of b allele frequency plots, when the recommended Roche DNA extraction kit was used instead of the Qiagen DNA extraction kit. Of great importance, we found that the QC-PCR provided an accurate prediction of chip quality as determined by comparing chip call rates with PCR signals derived from proprietary primer sets (rho= 0.6242, p<0.0001). The selection of restored DNA for future studies will be guided by the results from the QC-PCR assay.

These preliminary data demonstrate the promise of Illumina’s DNA restoration protocol for FFPE extracted DNA from tissues older than three decades. Further studies are required to determine the full potential of this method for SNP-based CGH analysis of large FFPE tumor banks.

